# Caspase-9: structure, mechanisms and clinical application

**DOI:** 10.18632/oncotarget.15098

**Published:** 2017-02-04

**Authors:** Ping Li, Libin Zhou, Ting Zhao, Xiongxiong Liu, Pengcheng Zhang, Yan Liu, Xiaogang Zheng, Qiang Li

**Affiliations:** ^1^ Institute of Modern Physics, Chinese Academy of Sciences, Lanzhou, People's Republic of China; ^2^ Key Laboratory of Heavy Ion Radiation Biology and Medicine of Chinese Academy of Sciences, Lanzhou, People's Republic of China; ^3^ Key Laboratory of Basic Research on Heavy Ion Radiation Application in Medicine, Gansu Province, Lanzhou, People's Republic of China; ^4^ University of Chinese Academy of Sciences, Beijing, People's Republic of China

**Keywords:** caspase-9, apoptosis, phosphorylation, alternative splicing, iCasp9

## Abstract

As the most intensively studied initiator caspase, caspase-9 is a key player in the intrinsic or mitochondrial pathway which is involved in various stimuli, including chemotherapies, stress agents and radiation. Caspase-9 is activated on the apoptosome complex to remain catalytic status and is thought of involving homo-dimerization monomeric zymogens. Failing to activate caspase-9 has profound physiological and pathophysiological outcomes, leading to degenerative and developmental disorders even cancer. To govern the apoptotic commitment process appropriately, plenty of proteins and small molecules involved in regulating caspase-9. Therefore, this review is to summarize recent pertinent literature on the comprehensive description of the molecular events implicated in caspase-9 activation and inhibition, as well as the clinical trials in progress to give deep insight into caspase-9 for suppressing cancer. We hope that our concerns will be helpful for further clinical studies addressing the roles of caspase-9 and its regulators demanded to identify more effective solutions to overcome intrinsic apoptosis-related diseases especially cancer.

## INTRODUCTION

**Figure 1:**
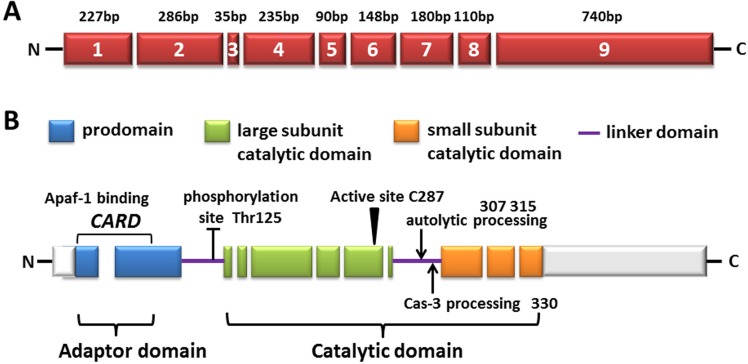
The diagram of human caspase-9 **A**. Exons are numbered inside the boxes and their lengths are shown on top. **B**. The predicted amino acid structure is depicted with the translated regions marked with different colors. Exon regions transcribed but not translated are depicted as white boxes. The three major domains are shaded: the pro-domain (within which the CARD domain is located, also called adaptor domain), the large subunit catalytic domain (LSCD), as well as the small subunit catalytic domain (SSCD, both LSCD and SSCD are also called catalytic domain). The linker domain (LD) is also indicated. The location of amino acid residues involved in the proteolytic processing of the procaspase-9 molecule is shown as well as the sites targeted for post-translational modification.

The two best-described apoptotic pathways existing in mammals—the extrinsic or receptor-induced pathway and intrinsic or mitochondrial stress-induced pathway—both use the caspases cascade. The two apoptosis pathways achieve the final commitment of physical dissolution of cellular structures by executioner proteases known as caspases, which are typically subdivided into initiator and effector caspases according to their placement in the apoptotic signal transduction. Procaspases are synthesized as single-chain inactive zymogens and should be activated to facilitate cell death. Procaspase-9 (Figure 1A), as the initiator caspase of the intrinsic apoptosis pathway, exists as monomers and possesses a long prodomain, which contains a caspase activation domain (CARD) motif, through which caspase-9 is recruited and activated at a multiprotein platform and then breaks up cells into apoptotic bodies [1]. Owing to its crucial function of converting the death signal to the first proteolytic event and directly mediating the activation of the lethal executioner protease, the manipulation of upstream events, especially the control of caspase-9, is a promising therapeutic goal for both proliferative and degenerative diseases.

## STRUCTURE AND ACTIVATION OF CASPASE-9

As previously mentioned, the CARD motif at the N-terminus of the long prodomain in caspase-9, can selectively bind to the CARD in Apaf-1 CARD through homotypic interactions [2]. Following it, a linker loop connects the prodomain and catalytic domain, which consists of large and small subunits (Figure 1B). The activity of caspase-9 is stimulated by dimerization instead of cleavage, although caspase-9 showed complete activity in its uncleaved form, probably owing to the long linker loop between subunits. Due to its length and function of connecting the large and small subunits, as its recruitment and dimerization within the apoptosome, the linker loop is presumed to move and gain access to the active site without cleavage [1, 3, 4]. Dimerization of caspase-9 leads to rapid autocatalytic cleavage, producing caspase-9 (p35/p12) [3, 5].

There are two hypotheses proposed for the activation of caspase-9. One is the “induced conformation model,” which represents that Apaf-1 apoptosome plays a function to alter conformation of caspase-9 via binding, which is required for activating caspase-9 [6, 7]. Apaf-1 is known to interact with caspase-9 through respective CARD domains [2, 8]. The crystal structure of a 1: 1 complex between the CARD domains of Apaf-1 and caspase-9 proves an indispensable complementary interface for caspase-9 activation [2]. Recently, Hu et al. provides strong evidence to support this model [9]. They demonstrated that a structure of a multimeric interaction between the CARD domains of Apaf-1 and caspase-9, which demands three kinds of interfaces rather than the above described 1:1 interaction, underlies caspase-9 activation [9]. The other hypothesis, the “induced proximity model,” supposes that the apoptosome supplies caspase-9 of a platform for its dimerization [10]. In this model, binding to the seven-membered recruitment platform apoptosome leads to an induced proximity of caspase-9. To retain the catalytic activity, caspase-9 has to maintain the binding to the apoptosome [4, 11, 12]. An evolved version, called proximity-induced dimerization, represents that the apoptosome mainly serves as a platform to accumulate the local concentration of procaspase-9 and promote its dimer-driven activation rather than as the trigger of conformational change [3, 7, 10]. Recent studies demonstrate that procaspase-9 is more affinitive with the apoptosome in contrast to its cleaved form [4, 13]. Additionally, procaspase-9 autoprocessing is not designed to activate caspase-9, but to turn on a molecular timer that stimulates the duration of apoptosome activity [13].

Although these new findings are, to some extent, conflicting and controversial, they promote the advancement of our cognition in the actual mechanism controlling caspase-9 activation and activity.

## CASPASE-9 EXPRESSION UNDER PHYSIOLOGICAL AND PATHOPHYSIOLOGICAL CONDITIONS

The constitutive expression of caspase-9 is ubiquitous in various mammalian tissues, and abnormal suppression of caspase-9 activity may lead to developmental aberrance. Genetic knockout studies demonstrate that the mice lacking caspase-9 die in the perinatal with serious abnormalities of the brain due to suppression of apoptosis during brain development [14, 15]. Caspase-9 null embryonic stem cells and embryonic fibroblasts are insensitive to the apoptotic stress induced by UV and γ-irradiation as well as dexamethasone [14, 15]. Besides the function it plays to decide cell survival during development and cell resistance to UV and γ-irradiation, there is also evidence that caspase-9 initiated apoptosis is believed to reflect the susceptibility of cancer cells to chemotherapy drugs. In detail, the acquired resistance of head and neck squamous cell carcinoma cells to cisplatin may attribute to the reduced caspase-9 activity and Apaf-1 expression [16]. Mueller and colleagues found that failure of caspase-9 activation increases the apoptotic threshold induced by cisplatin in testicular cancer cells, although it could be overcome by increasing the dose of cisplatin [17]. Furthermore, the observed correlation of high p53 expression and caspase-9 inhibition has been shown to increase the resistance of tumor cells to cisplatin treatment [18]. These data signify that caspase-9 suppression might be a tumor escape mechanism of apoptosis when cytoplasmic p53 locally accumulates. However, unexpected evidence derived from Tamaki et al. indicated that the treatment to prostate cancer cells with inhibited caspase-9 using ABT-263 could trigger apoptosis mainly through activation of caspase-8 [19]. The paradox is that because caspase-9 is activated by ABT-263 in PC3 cells, how does inhibition of caspase-9 enhance the apoptosis induced by ABT-263 in a caspase-8-dependent manner? Until recently, no reasonable explanation for this phenomenon was available, and further study is needed to examine the precise mechanism. Based on the above studies, the destiny of cells to decide apoptosis and survival is not only determined by caspase-9 but also by the relative abundance and interactions of various crucial apoptotic proteins. Actually, it has been demonstrated that the expression level of caspase-9 and the associated proteins in the intrinsic apoptosis pathway can serve as promising response markers and predict the therapeutic effect of 5-fluorouracil-based chemotherapy [20]. In addition, the functional polymorphisms of the CASPASE-9 gene and subsequent corruption of the intrinsic apoptosis pathway has been shown to participate in tumor susceptibility to lung, bladder, pancreatic, colorectal and gastric cancers [21–25]. Some studies have shown that dysfunctional apoptosome not only conduces to carcinogenesis but is associated to various degenerative disorders [26]. Andreoli et al. [27] stated the correlation between CASPASE-9 gene locus polymorphisms and numerous sclerosis, manifesting that the CASPASE-9 (Ex5 + 32G/A) GG genotype may be involved in a higher risk of numerous sclerosis. It has also been reported that the expression levels of CASPASE-9 are remarkably improved in the degenerated disc, and the CASPASE-9-1263A/G polymorphism is related to a higher risk of discogenic low back pain [28]. Activated caspase-9 and caspase-3 activities are present only at the endstage of Huntington's disease, suggesting that apoptosis may contribute to neuronal death at the endstage of disease [29]. The evidence above proves that caspase-9 is required for maintaining cell homeostasis via the cleavage of multiple key players implicated in apoptosis. Caspase-9 is essential to eliminate cells by executing apoptotic death early in development stage as it is indispensable to inhibit proliferative diseases through the continuous removal of irreparable cells in the lifecycle.

Additionally, Murray and colleagues provide important implications that the function of caspase-3 and caspase-9 are not limited in cellular disassembly, the proteases also participate to determine the destiny of myoblast differentiation [30]. Signals resulting in caspase-3 activation are generally thought of executing cell death. Thus, their data adds to our current knowledge. Recently, it has been reported knockdown of caspase-9 may have genuine potential in the treatment of bovine skeletal muscle atrophy [31]. It is very important to screen out the caspase substrates that are cleaved in differentiating myoblasts and to make clear how the intrinsic apoptotic pathway is used to complete myotube formation.

Given the crucial role of caspase-9 under physiological and pathophysiological conditions, it is natural to believe that a variety of proteins and small molecules are implicated in regulating its function. The endogenous regulators of caspase-9 are summarized and discussed as follows.

## ENDOGENOUS REGULATORS OF CASPASE-9

**Figure 2:**
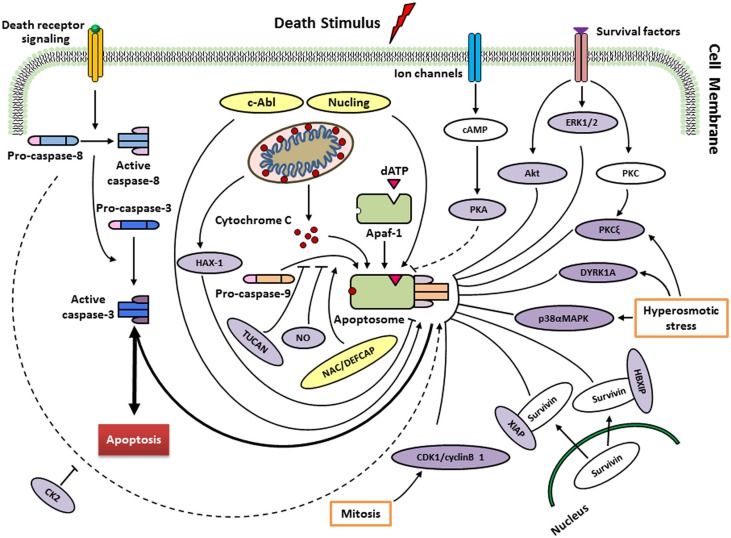
Regulation of caspase-9 by endogenous regulators in different signalling pathways Following cytochrome c release from mitochondria, a heptameric wheel-like multimeric complex, the apoptosome, induced and composed by Apaf-1 and procaspase-9. Factors such as ATP, can inhibit this process by directly inhibiting the interaction between Apaf-1 and cytochrome c. Recruitment of procaspase-9 to the apoptosome is antagonized by TUNCAN, and stimulated by NAC/DEFCAP. HAX-1 may inhibit caspase-9 activation, thereby suppressing apoptosis in cardiac myocytes. Direct phosphorylation at Thr125 by ERK1/2 (growth/survival signals), CDK1-cyclin B1 (in mitosis), p38αMAPK (upon hyperosmotic stress) and DYRK1A (in development and upon hyperosmotic stress) inhibits caspase-9 activity. PKCξ also inhibits phosphorylation of caspase-9 at Ser144 induced by hyperosmotic stress. Akt/PKB, as a protein kinase, suppresses the activation of caspase-9 in response to extracellular growth/survival signals. The interactions of HBIXP and XIAP with survivin block the activation of caspase-9 through distinct mechanisms. PKA seems to block the recruitment of caspase-9 to apoptosome rather than inhibitory effect on caspase-9 activation (unclear). CK2 phosphorylates caspase-9 at Ser348 to protect from caspase-8 cleavage in mouse, but not conserved in humans. Nitrosylation of caspase-9 by the donor of NO suggests cleavage inhibition of procaspase-9 and consequently apoptosis. Conversely, phosphorylation at Tyr153 by c-Abl stimulates activiation of caspase-9. Nucling recruits and transports the apoptosome when responding to the apoptosis induced by stresses. See text for more details.

To ensure that apoptosis occurs appropriately, the intrinsic apoptotic pathway is controlled at various stages. Human caspase-9 was first discovered to be endogenously inhibited two decade ago [32], then it has continuously been argued to be the target of multiple proteins and small molecules that respond to extracellular signals and cellular stresses (Figure 2; Table 1). As a well-known inhibitory site in caspase-9, the cleavage of procaspase-9 is inhibited by ERK1/2, DYRK1A, CDK1-cylinB1 and p38α at the Thr125 phosphorylation site. Thr125 is in the hinge region close to the N-terminus of the large subunit, but its phosphorylation does not inhibit the recruitment of caspase-9 to Apaf-1 based on a report of Allan et al. [33]. It seems that the phosphorylated caspase-9 may serve as a dominant negative regulator, adjusting the recruitment of non-phosphorylated caspase-9 to the apoptosome platform [34]. Nevertheless, the exact mechanism behind the suppression of caspase-9 activity at the phosphorylation site of Thr125 remains obscure.

**Table 1:** Endogenous regulators of caspase-9

Efficacy	Factor	Target	Mechanism of action	Reference(s)
Inhibition	ERK2	Caspase-9 (Thr125-P)	Inhibiting caspase-9 processing	[33, 58]
	DYRK1A	Caspase-9 (Thr125-P)	Inhibiting caspase-9 processing	[59–61]
	CDK1/cyclin B1	Caspase-9 (Thr125-P)	Inhibiting caspase-9 processing	[62]
	P38α MAPK	Caspase-9 (Thr125-P; most likely indirect)	Inhibiting caspase-9 processing	[60]
	Akt/PKB	Caspase-9 (Ser196-P)	Inhibiting caspase-9 processing	[32]
	PKCξ	Caspase-9 (Ser144-P)	Inhibiting caspase-9 processing	[36]
	PKA	Caspase-9(Ser99-P, Ser183-P, Ser195-P, but not required)	Inhibiting caspase-9 recruitment to the apoptosome? Unclear.	[37]
	CK2	Caspase-9 (Ser302-P, Ser307-P and Ser310-P) unclear	Inhibiting caspase-9 processing in murine cells. Unclear in human cells.	[38]
	XIAP	Caspase-9	Inhibiting processed caspase-9	[42, 43, 63, 64]
	TUCAN	Caspase-9	Inhibiting Apaf-1–caspase-9 interaction	[44]
	HBXIP	Caspase-9	Inhibiting Apaf-1–caspase-9 interaction as HBXIP–survivin complex	[45]
	HAX-1	Caspase-9	Inhibiting caspase-9 activity	[46, 47]
	NO	Caspase-9	Inhibiting caspase-9 activity by nitrosylation	[48, 49, 65]
Enhancement	c-Abl	Caspase-9	Enhancing caspase-9 processing	[41, 52]
	Nucling	Caspase-9	Enhancing apoptosome stability; transports to nucleus	[66]
	NAC/DEFCAP	Apaf-1 and Caspase-9	Enhancing caspase-9 recruitment to the apoptosome; mechanism unclear.	[53, 54]

In addition to its phosphorylation of Thr125, Cardone et al. [32] reported protein kinase B (PKB, also called Akt) phosphorylates caspase-9 at Ser196. The protein activity of recombinant caspase-9 is inhibited by Akt through phosphorylation at Ser196 *in vitro*. However, when a mutant procaspase-9 (Ser196Ala) is used, Akt-resistant apoptosis is induced. Ser196 is not a conserved site in rodents, and Akt is unable to phosphorylate murine caspase-9 *in vitro* [35]. Therefore, the role of PKB in caspase-9 regulation needs to be clarified.

Human caspase-9 is also inhibited by an isoform of protein kinase C (PKCξ) at the phosphorylation site Ser144 in a cell-free system and when cells respond to protein phosphatase inhibitor okadaic acid [36]. The inhibition of PKCξ and mutation of caspase-9 fail to phosphorylate Ser144 and facilitate caspase-3 activation. Hyperosmotic stress also inhibits caspase-9 via the phosphorylation of Ser144 and activates PKC. In addition, hyperosmolarity is found to disrupt the binding of PKCξ to caspase-9 in non-stressed cells. Nevertheless, phosphorylation of caspase-9 at Ser144 is unnecessary for the dissociation of PKCξ, suggesting that other underlying mechanisms adjust their dynamic association [36].

Three phosphorylation sites of Ser99, Ser183 and Ser195 are involved in the suppression of caspase-9 activity by protein kinase A (PKA) in Xenopus egg extracts and in a human cell-free system. However, mutational analysis discovered that PKA prevents caspase-9 from recruiting to the apoptosome rather than inhibiting caspase-9 activation [37].

Casein kinase 2 (CK2) enable caspase-9 to be phosphorylated at Ser348 site in murine cells (equivalent to human Ser310), blocking its cleavage at Asp353 by caspase-8, which is irrelevant to cytochrome c-mediated activation of caspase-9, incurred by tumor necrosis factor-α [38]. Mass spectrometric analyses have identified a batch of phosphorylation sites of caspase-9 in the murine protein [39] and the human protein [40]. However, whether CK2 answers for the phosphorylation of human caspase-9 at Ser348 awaits confirmation [41].

XIAP belongs to the IAP protein family. Biochemical and structural techniques reveal that XIAP and caspase-9 compose a complex to block the dimerization of the protease, via the binding interaction of the BIR3 domain of XIAP to the neo-N-terminus of the small subunit of caspase-9 [42, 43]. TUCAN is observed to interfere with the binding of Apaf-1 to caspase-9 through binding to caspase-9, and inhibits the activation of the protease induced by cytochrome c, which is the activator of Apaf-1. Survivin-HBXIP complex, rather than the individual proteins, binds to procaspase-9, refraining from its recruitment to Apaf-1, thus selectively inhibits apoptosis occurred through the mitochondria pathway [45].

HS-1-associated protein-1 (HAX-1) interacts with caspase-9 when localizes to the mitochondria. Using a cell-free caspase activation assay, caspase-9 activation is found to be suppressed in a dose-dependent manner by recombinant HAX-1 protein. HAX-1 silence by corresponding siRNA leads to severe apoptosis in adult cardiac myocytes. Upon apoptotic stimuli, some caspase-9 is discovered to move to the mitochondria and co-localized with HAX-1 [46]. Recent study also displayed that apoptosis in prostate cancer is suppressed by inhibition of caspase-9 [47]. Thus, HAX-1 is an anti-apoptotic molecule through inhibiting caspase-9 in some special cells.

Nitrosylation appears to an additional mechanism regulating caspase-9 activity. This involves the physiological effect of nitric oxide (NO) on the cells. Analysis of NO expression in human cholangiocarcinoma cells, as well as human monocytes, revealed a marked decrease in caspase-9 activity in the presence of pharmacological NO donors [48, 49]. As NO can evoke a pro-apoptotic or an anti-apoptotic response depending on cell type and NO levels [50], caspase nitrosylation may ultimately reflect the local cellular microenvironment, including, but not limited to the overall redox conditions encountered [51].

The c-Abl tyrosine kinase also binds directly to caspase-9 in response to stress stimuli [52]. Caspase-9 is phosphorylated at Tyr-153 *in vitro* by c-Abl upon DNA damaging agents. In addition, c-Abl inhibitor, STI571, prevents caspase-9 from autoprocessing induced by DNA damage to the p35 subunit and the downstream activation of caspase-3. Based on these findings, c-Abl may enhance caspase-9 autoprocessing when responding to DNA damage stresses, although the general mechanism of Tyr153 phosphorylation regarding the regulation of caspase-9 stays clarified [41]. Similarly, NAC/DEFCAP and nucling are also thought to enhance caspase-9 function [53, 54]. It remains obscure how this is achieved, but it will be meaningful to explore the possible mechanisms that activate and sustain the catalytic function of caspase-9.

MicroRNAs (miRNAs) comprise small non-coding regulatory RNA molecules engaged in regulating several crucial cellular processes, like apoptosis, development and differentiation. Because of their concerning to the apoptosis regulation, identified miRNAs implicated in targeting caspase-9, are reviewed below (Table 2).

**Table 2:** MicroRNAs regulating caspase-9

Factor	Target	Mechanism of action	Reference(s)
miRNA-24a	Apaf-1 and caspase-9	Inhibiting caspase-9 and Apaf-1 activities	[55]
miRNA-582-5p	Caspase-9	Inhibiting caspase-9 expression at both protein and mRNA levels	[56]
miRNA-23a	Caspase-9	Inhibiting caspase-9 activity	[57]

Until now, only three miRNAs, including miRNA-24a, miRNA-582-5p and miRNA-23a are found to suppress caspase-9 activation, resulting in a block in mitochondrial apoptosis. Reporter assays and loss-of-function experiments revealed that the loss of miRNA-24a function leads to an increase in caspase-9 protein levels without altering mRNA levels, and the miRNA knockdown phenotype is dependent on the function of caspase-9. Thus, miRNA-24a is an essential regulator inhibitor of caspase-9 and programmed cell death [55]. After microarray analysis and validation in a variety of glioblastoma cell lines and human specimens, Floyd et al. screened and demonstrated that miRNA-582-5p targets caspase-3 and caspase-9, and effectively inhibited caspase-9 protein and mRNA expression [56]. When colon cancer cells were treated with anti-carcinogen 5-fluorouracil, increased expression of miRNA-23a inhibited Apaf-1 expression levels. Apaf-1 is identified for the first time as a target of miRNA-23a and enhancement of caspase-9 activation is induced by miRNA-23a antisense [57].

Caspase-9 represents a pivotal signaling element governing the apoptotic commitment process, and the regulatory features for discrete modulation of caspase-9 activity by a wide range of intra- and inter-molecular events determine the essential role of caspase-9 in maintaining appropriate cellular homeostasis.

## ALTERNATIVE TRANSCRIPTS OF CASPASE-9 AND MECHANISTIC REGULATION

Alternative splicing (AS) of pre-messenger RNAs is a major process that contributes to the transcriptome and proteome diversity of the human genome. It acts on essential physiological processes in human development and has also been found to be associated with various diseases, including cancer [67]. Splicing executes through the concerted cooperations of multi-subunit complexes. The process of splicing regulation is generally directed by cis-acting elements and trans-acting RNA-binding proteins (RBP). Two well-known families belong to RBP are serine/arginine-rich (SR) proteins and heterogeneous nuclear ribonucleoproteins (hnRNP).

**Figure 3:**
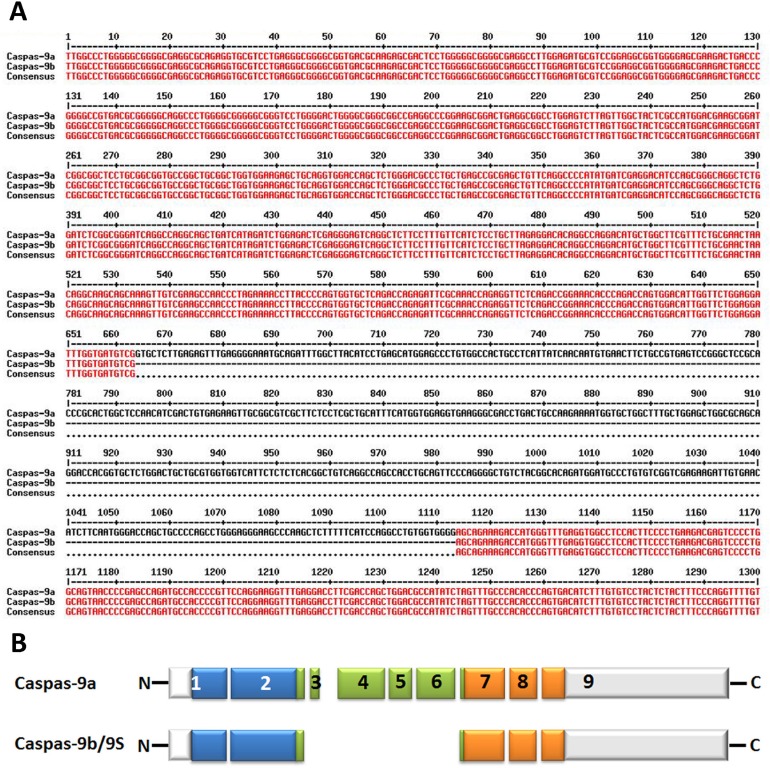
Alternative splice variants of human caspase-9 **A**. Nucleotide alignment of human caspase-9a with caspase-9b. The bases marked with red color show the identical sequence of both caspase-9a and caspase-9b while the single-line bases in black color represent the alternative splicing sites. The alignment is processed by the online multiple-sequence alignment software MultAlin. **B**. Deduced structural comparison of the human caspase-9a and caspase-9b is presented. The two sequences refer to the Refseq. NM_001229.4 (caspase-9a) and NM_001278054.1 (caspase-9b). As indicated in the diagram, caspase-9b/9S lacks the entire large subunit catalytic domain (LSCD) found in the full-length protein. The aligned amino acid sequences are depicted with the translated regions marked with different colors. Exon regions transcribed but not translated are depicted as white boxes.

The splicing of caspase-9 gene generates two isoforms caspase-9a and caspase-9b/9S, which can be generated by the inclusion or exclusion of a 4-exon cassette including exon 3, 4, 5, and 6 in the mature caspase-9 mRNA (Figure 3A&3B). Caspase-9a bears the full cysteine protease activity, whereas caspase-9b is short of the central catalytic domain (Figure 1B & Figure 3B) [68, 69]. Caspase-9b functions as an apoptotic inhibitor by against caspase-9a for attaching to the apoptosome and consequently suppressing caspase enzyme cascade [70]. Therefore, caspase-9b functions as an endogenous dominant-negative isoform. Specifically, Chalfant and colleagues revealed that the ratio of caspase 9a/9b mRNA is pretty lower in a large proportion of human NSCLC tumors. A low caspase 9a/9b ratio, demanded for the tumorigenecity of NSCLC cells, is correlated with an anti-apoptotic phenotype [71, 72].

Several factors are involved in the AS of caspase-9 in some NSCLCs, including endogenous ceramides [73], SRSF1 (SRp30a or ASF/SF2) [72, 74, 75], hnRNP L and hnRNP U [70, 71]. De novo ceramide synthesis and SRSF1 have been reported to decrease the level of caspase-9b mRNA, which represses the inclusion of the 4-exon cassette to favor the formation of caspase-9b. In contrast, the activation of hnRNP L lowers the caspase-9a/9b ratio in NSCLC cells. Significantly, down-regulation of SRSF1 or phosphorylation of hnRNP L and the following rise of caspase-9a/9b mRNA ratio have been shown to deprive NSCLC cells of tumorigenic capacity and enhance their sensitivity to chemotherapeutics through the AS of caspase-9 [70, 71, 75]. As a limiting factor for caspase-9b formation, hnRNP U competes with hnRNP L for binding to C9/E3 to determine the induction of caspase-9b expression [70]. Taken together, these studies strongly suggest a novel and crucial distal mechanism in NSCLC and offer promise of a new target for the development of therapeutics in the defense against cancers [72].

## INDUCIBLE CASPASE-9 SUICIDE GENE AND ITS APPLICATION

**Figure 4:**
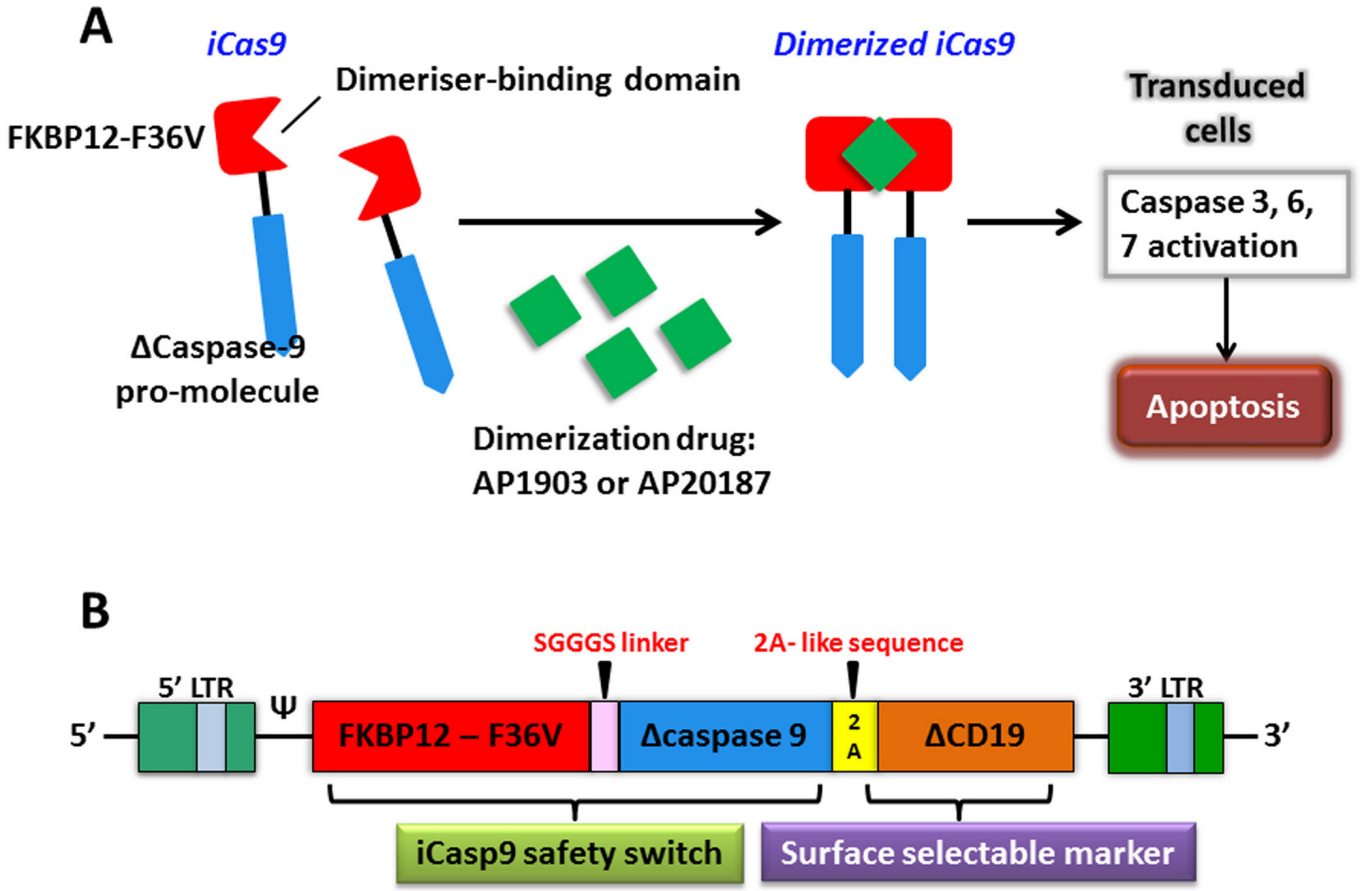
The apoptosis of transduced cells incurred by activated iCasp9 **A**. iCasp9 is formed by connecting a drug-binding domain, FKBP12-F36V with Δcaspase9 via a SGGGS linker. Binding of small CID (AP1903 or AP20187) leads to dimerization of iCas9, thereby relaying activation signals and initiating mitochondrial apoptosis pathway. **B**. The bicistronic transgene, iCaps9.2A.ΔCD19, comprises iCasp9 sequence, linked via a short 2A-like sequence, with truncated CD19 using as a selectable marker.

The expression of chimeric antigen receptor (CAR) by genetic engineering can provide a new way for tumor immunotherapy. The inducible caspase-9 (iCasp9) provides a regime for the elimination of CAR T cells without appropriate activation, which has been proven by successful clinical trials as a novel suicide gene [76]. The optimized iCasp9 molecule is based on a drug-binding domain, FK506-F36V, which is linked by a short Ser-Gly-Gly-Gly-Ser linker to ΔCaspase9, a caspase-9 without its physiological CARD. AP1903 or AP20187, as a chemical inducer of dimerization (CID) binds to iCasp9 and activates the dimerization of ΔCaspase9, which leads to the activation of downstream caspase cascade, resulting in apoptosis (Figure 4A). An administration of 10 nM dose of CID can produce apoptosis in >99% of cells with high expression of iCasp9 only in one hour [77]. There are several advantages of the system that over current or previously available suicide genes, such as the herpes simplex virus thymidine kinase (HSV-TK) gene, the E. coli-derived cytosine deaminase gene, the CD20 gene, the death effector domain of FADD, and the effector caspases [78–84]. It manifests as a good balance among low potential immunogenicity of human gene products, low dimerizer-irrelevant activity and high sensitivity to dimerizer-induced apoptotic death.

The iCasp9 safety switch has been clinically verified in haploidentical hematopoietic stem cell transplantation (haplo-HSCT). Haplo-HSCT matches only 1/2 to 4/5 human leukocyte antigen (HLA) loci and is implicated in a high rate of fatal acute graft-versus-host disease (GVHD) if no specific steps are taken [85]. In a pre-clinical study of this system, an *in vivo* severe combined immunodeficiency (SCID) mouse-human xenograft model was investigated where the administration dose of CID killed more than 99% circulating human GFP^+^ T cells in three days. What's more, just after half an hour of CID incubation, killing effects via iCasp9 were found to induce a rapid and high proportion of early apoptotic Annexin V^+^ cells [86]. In order to reduce the risk of GVHD produced by donor T-cells haplo-HSCT in recipients and promote immune reconstitution, Tey et al. [87] generated a gammaretroviral vector encoding the iCaps9.2A.ΔCD19 cassette composing of iCasp9 joined, by a 2A-like linker, to ΔCD19 to transduce allodepleted T cells (Figure 4B). CARD was deleted from the CASPASE-9 sequence and replaced with FKBP12 to improve transgene activity. ΔCD19, as a surface selectable marker, makes sure most of genetically engineered T cells carry iCasp9 switch [85, 87]. A study has offered strong support that this iCasp9-based conditional safety switch can rapidly remove T cells and prevent an ongoing autoimmune attack in a murine model [88]. Hoyos et al. [89] have improved CD19-CAR T technology by generating a novel construct that co-expresses interleukin-15 (IL-15) and the iCasp9 suicide gene. Performance improvements of iC9/CAR.19/IL-15^+^ T cells are tremendously improved, particularly in numeric expansion upon antigen stimuli and improved anti-tumor effects *in vivo*. In a clinical trial study, five recipients at the ages of 3 to 17 who had experienced stem-cell transplantation for relapsed acute leukemia were treated using infusions of iCasp9-expressing donor T cells. The iCasp9 suicide switch removed more than 90% of engineered T cells only half an hour following the administration of a single dose of CID in four patients who has GVHD, and terminated GVHD without reoccurrence [76]. The long-term follow-up (3.5 years) of all ten patients enrolled in this trial showed that iC9-T cell infusions and the administration of CID effectively helped patients to reconstruct immune system after the haplo-HSCT and prevented the patients from pathogenic infections [90].

As CAR T-cell therapies have shown exciting promise in the clinic, so have its serious side-effects. The iCasp9 suicide gene technology has demonstrated superiority at improving the safety profile of CAR T-cell technology and expanding its scope of application in the clinic.

## CONCLUSION AND PERSPECTIVE

Despite recent advances in understanding the roles that caspase-9 plays in the tumorigenic capacity of human NSCLC and in CAR T therapy trial, a number of questions still remain concerning its precise mechanism of activation/formation. For example, we do not yet understand the precise dynamics of caspase-9 activation and activity, nor do we understand how caspase-9 is activated for non-apoptotic purposes without killing cells. Studies of caspase-9 as not only an agent of cellular disassembly but as an agent that helps to determine the fate of differentiating myoblasts has challenged our common understanding of caspase-9 function [30]. This finding suggests the presence (at least in specific cell types) of a mechanism of caspase-9 activation irrelevance to cytochrome c.

Emerging evidence that caspase-9 may be altered under various pathological conditions raises the exciting possibility that caspase-9 may act as a therapeutic. Because the inhibition of caspase-9 activity might serve as acquired chemotherapeutic resistance in specific types of human cancer cell lines [16, 17, 91, 92], the requirement of DNA damage-induced neural precursor cell death involves caspase-9 but is not associated with a detectable loss of cytochrome c from mitochondria, indicating that caspase-9 can act directly as an apoptotic effector molecule [93]. The selective induction of apoptosis in cancerous cells has been a long-term goal of researchers against cancer. Thus, continued studies of caspase-9 activation, function and regulation may soon make caspase-9 a viable therapeutic target not only for cancer treatment but also for degenerative and developmental disorders.

## References

[1] Bratton SB, Salvesen GS (2010). Regulation of the Apaf-1-caspase-9 apoptosome. Journal of Cell Science.

[2] Qin H, Srinivasula SM, Wu G, Fernandes-Alnemri T, Alnemri ES, Shi Y (1999). Structural basis of procaspase-9 recruitment by the apoptotic protease-activating factor 1. Nature.

[3] Renatus M, Stennicke HR, Scott FL, Liddington RC, Salvesen GS (2001). Dimer formation drives the activation of the cell death protease caspase 9. Proceedings of the National Academy of Sciences.

[4] Stennicke HR, Deveraux QL, Humke EW, Reed JC, Dixit VM, Salvesen GS (1999). Caspase-9 Can Be Activated without Proteolytic Processing. Journal of Biological Chemistry.

[5] Würstle ML, Laussmann MA, Rehm M (2012). The central role of initiator caspase-9 in apoptosis signal transduction and the regulation of its activation and activity on the apoptosome. Experimental Cell Research.

[6] Shi Y (2004). Caspase Activation: Revisiting the Induced Proximity Model. Cell.

[7] Chao Y, Shiozaki EN, Srinivasula SM, Rigotti DJ, Fairman R, Shi Y (2005). Engineering a Dimeric Caspase-9: A Re-evaluation of the Induced Proximity Model for Caspase Activation. PLoS Biol.

[8] Li P, Nijhawan D, Budihardjo I, Srinivasula SM, Ahmad M, Alnemri ES, Wang X (1997). Cytochrome c and dATP-Dependent Formation of Apaf-1/Caspase-9 Complex Initiates an Apoptotic Protease Cascade. Cell.

[9] Hu Q, Wu D, Chen W, Yan Z, Yan C, He T, Liang Q, Shi Y (2014). Molecular determinants of caspase-9 activation by the Apaf-1 apoptosome. Proceedings of the National Academy of Sciences of the United States of America.

[10] Boatright KM, Salvesen GS (2003). Mechanisms of caspase activation. Current Opinion in Cell Biology.

[11] Rodriguez J, Lazebnik Y (1999). Caspase-9 and APAF-1 form an active holoenzyme. Genes & Development.

[12] Bratton SB, Walker G, Srinivasula SM, Sun XM, Butterworth M, Alnemri ES, Cohen GM (2001). Recruitment, activation and retention of caspases-9 and -3 by Apaf-1 apoptosome and associated XIAP complexes. The EMBO Journal.

[13] Malladi S, Challa-Malladi M, Fearnhead HO, Bratton SB (2009). The Apaf-1·procaspase-9 apoptosome complex functions as a proteolytic-based molecular timer. The EMBO Journal.

[14] Hakem R, Hakem A, Duncan GS, Henderson JT, Woo M, Soengas MS, Elia A, de la Pompa JL, Kagi D, Khoo W, Potter J, Yoshida R, Kaufman SA (1998). Differential Requirement for Caspase 9 in Apoptotic Pathways In Vivo. Cell.

[15] Kuida K, Haydar TF, Kuan C-Y, Gu Y, Taya C, Karasuyama H, Su MSS, Rakic P, Flavell RA (1998). Reduced Apoptosis and Cytochrome c-Mediated Caspase Activation in Mice Lacking Caspase 9. Cell.

[16] Kuwahara D, Tsutsumi K, Oyake D, Ohta T, Nishikawa H, Koizuka I (2003). Inhibition of caspase-9 activity and Apaf-1 expression in cisplatin-resistant head and neck squamous cell carcinoma cells. Auris Nasus Larynx.

[17] Mueller T, Voigt W, Simon H, Fruehauf A, Bulankin A, Grothey A, Schmoll HJ (2003). Failure of Activation of Caspase-9 Induces a Higher Threshold for Apoptosis and Cisplatin Resistance in Testicular Cancer. Cancer Research.

[18] Chee JL, Saidin S, Lane DP, Leong SM, Noll JE, Neilsen PM, Phua YT, Gabra H, Lim TM (2013). Wild-type and mutant p53 mediate cisplatin resistance through interaction and inhibition of active caspase-9. Cell Cycle.

[19] Tamaki H, Harashima N, Hiraki M, Arichi N, Nishimura N, Shiina H, Naora K, Harada M (2014). Bcl-2 family inhibition sensitizes human prostate cancer cells to docetaxel and promotes unexpected apoptosis under caspase-9 inhibition. Oncotarget.

[20] Hector S, Rehm M, Schmid J, Kehoe J, McCawley N, Dicker P, Murray F, McNamara D, Kay EW, Concannon CG, Huber HJ, Prehn JH (2012). Clinical application of a systems model of apoptosis execution for the prediction of colorectal cancer therapy responses and personalisation of therapy. Gut.

[21] Park JY, Park JM, Jang JS, Choi JE, Kim KM, Cha SI, Kim CH, Kang YM, Lee WK, Kam S, Park RW, Kim IS, Lee JT (2006). Caspase 9 promoter polymorphisms and risk of primary lung cancer. Human Molecular Genetics.

[22] Gangwar R, Mandhani A, Mittal RD (2009). Caspase 9 and Caspase 8 Gene Polymorphisms and Susceptibility to Bladder Cancer in North Indian Population. Annals of Surgical Oncology.

[23] Theodoropoulos GE, Michalopoulos NV, Panoussopoulos SG, Taka S, Gazouli M (2010). Effects of Caspase-9 and Survivin Gene Polymorphisms in Pancreatic Cancer Risk and Tumor Characteristics. Pancreas.

[24] Liamarkopoulos E, Gazouli M, Aravantinos G, Tzanakis N, Theodoropoulos G, Rizos S, Nikiteas N (2011). Caspase 8 and caspase 9 gene polymorphisms and susceptibility to gastric cancer. Gastric Cancer.

[25] Theodoropoulos G, Gazouli M, Vaiopoulou A, Leandrou M, Nikouli S, Vassou E, Kouraklis G, Nikiteas N (2011). Polymorphisms of Caspase 8 and Caspase 9 gene and colorectal cancer susceptibility and prognosis. International Journal of Colorectal Disease.

[26] Sang T-K, Li C, Liu W, Rodriguez A, Abrams JM, Zipursky SL, Jackson GR (2005). Inactivation of Drosophila Apaf-1 related killer suppresses formation of polyglutamine aggregates and blocks polyglutamine pathogenesis. Human Molecular Genetics.

[27] Andreoli V, Trecroci F, A La Russa, Valentino P, Condino F, Latorre V, Nisticò R, Pirritano D, F Del Giudice, Canino M, Cittadella R, Quattrone A (2009). CASP-9: A susceptibility locus for multiple sclerosis in Italy. Journal of Neuroimmunology.

[28] Guo TM, Liu M, Zhang YG, Guo WT, Wu SX (2011). Association between Caspase-9 promoter region polymorphisms and discogenic low back pain. Connective Tissue Research.

[29] Kiechle T, Dedeoglu A, Kubilus J, Kowall N, Beal MF, Friedlander R, Hersch S, Ferrante R (2002). Cytochrome C and caspase-9 expression in Huntington's disease. NeuroMolecular Medicine.

[30] Murray TV, McMahon JM, Howley BA, Stanley A, Ritter T, Mohr A, Zwacka R, Fearnhead HO (2008). A non-apoptotic role for caspase-9 in muscle differentiation. Journal of Cell Science.

[31] Van Ba H, Hwang I (2014). Role of caspase-9 in the effector caspases and genome expressions, and growth of bovine skeletal myoblasts. Development, Growth & Differentiation.

[32] Cardone MH, Roy N, Stennicke HR, Salvesen GS, Franke TF, Stanbridge E, Frisch S, Reed JC (1998). Regulation of Cell Death Protease Caspase-9 by Phosphorylation. Science.

[33] Allan LA, Morrice N, Brady S, Magee G, Pathak S, Clarke PR (2003). Inhibition of caspase-9 through phosphorylation at Thr 125 by ERK MAPK. Nat Cell Biol.

[34] Riedl SJ, Salvesen GS (2007). The apoptosome: signalling platform of cell death. Nat Rev Mol Cell Biol.

[35] Fujita E, Jinbo A, Matuzaki H, Konishi H, Kikkawa U, Momoi T (1999). Akt Phosphorylation Site Found in Human Caspase-9 Is Absent in Mouse Caspase-9. Biochemical and Biophysical Research Communications.

[36] Brady SC, Allan LA, Clarke PR (2005). Regulation of Caspase 9 through Phosphorylation by Protein Kinase C Zeta in Response to Hyperosmotic Stress. Molecular and Cellular Biology.

[37] Martin MC, Allan LA, Lickrish M, Sampson C, Morrice N, Clarke PR (2005). Protein Kinase A Regulates Caspase-9 Activation by Apaf-1 Downstream of Cytochrome c. Journal of Biological Chemistry.

[38] McDonnell MA, Abedin MJ, Melendez M, Platikanova TN, Ecklund JR, Ahmed K, Kelekar A (2008). Phosphorylation of Murine Caspase-9 by the Protein Kinase Casein Kinase 2 Regulates Its Cleavage by Caspase-8. Journal of Biological Chemistry.

[39] Dai J, Jin WH, Sheng QH, Shieh CH, Wu JR, Zeng R (2007). Protein Phosphorylation and Expression Profiling by Yin-Yang Multidimensional Liquid Chromatography (Yin-Yang MDLC) Mass Spectrometry. Journal of Proteome Research.

[40] Dephoure N, Zhou C, Villén J, Beausoleil SA, Bakalarski CE, Elledge SJ, Gygi SP A quantitative atlas of mitotic phosphorylation. Proceedings of the National Academy of Sciences.

[41] Allan LA, Clarke PR (2009). Apoptosis and autophagy: Regulation of caspase-9 by phosphorylation. FEBS Journal.

[42] Shiozaki EN, Chai J, Rigotti DJ, Riedl SJ, Li P, Srinivasula SM, Alnemri ES, Fairman R, Shi Y (2003). Mechanism of XIAP-Mediated Inhibition of Caspase-9. Molecular Cell.

[43] Srinivasula SM, Hegde R, Saleh A, Datta P, Shiozaki E, Chai J, Lee RA, Robbins PD, Fernandes-Alnemri T, Shi Y, Alnemri ES (2001). A conserved XIAP-interaction motif in caspase-9 and Smac/DIABLO regulates caspase activity and apoptosis. Nature.

[44] Pathan N, Marusawa H, Krajewska M, Matsuzawa S, Kim H, Okada K, Torii S, Kitada S, Krajewski S, Welsh K, Pio F, Godzik A, Reed JC (2001). TUCAN, an Antiapoptotic Caspase-associated Recruitment Domain Family Protein Overexpressed in Cancer. Journal of Biological Chemistry.

[45] Marusawa H, Si Matsuzawa, Welsh K, Zou H, Armstrong R, Tamm I, Reed JC (2003). HBXIP functions as a cofactor of survivin in apoptosis suppression. The EMBO Journal.

[46] Han Y, Chen YS, Liu Z, Bodyak N, Rigor D, Bisping E, Pu WT, Kang PM (2006). Overexpression of HAX-1 Protects Cardiac Myocytes From Apoptosis Through Caspase-9 Inhibition. Circulation Research.

[47] Yan J, Ma C, Cheng J, Li Z, Liu C (2015). HAX-1 inhibits apoptosis in prostate cancer through the suppression of caspase-9 activation. Oncology reports.

[48] Török NJ, Higuchi H, Bronk S, Gores GJ (2002). Nitric Oxide Inhibits Apoptosis Downstream of Cytochrome c Release by Nitrosylating Caspase 9. Cancer Research.

[49] Zeigler MM, Doseff AI, Galloway MF, Opalek JM, Nowicki PT, Zweier JL, Sen CK, Marsh CB (2003). Presentation of Nitric Oxide Regulates Monocyte Survival through Effects on Caspase-9 and Caspase-3 Activation. Journal of Biological Chemistry.

[50] Dimmeler S, Zeiher AM (1997). Nitric Oxide and Apoptosis: Another Paradigm for the Double-Edged Role of Nitric Oxide. Nitric Oxide.

[51] Johnson CR, Jarvis WD (2004). Caspase-9 regulation: An update. Apoptosis.

[52] Raina D, Pandey P, Ahmad R, Bharti A, Ren J, Kharbanda S, Weichselbaum R, Kufe D (2005). c-Abl Tyrosine Kinase Regulates Caspase-9 Autocleavage in the Apoptotic Response to DNA Damage. Journal of Biological Chemistry.

[53] Chu ZL, Pio F, Xie Z, Welsh K, Krajewska M, Krajewski S, Godzik A, Reed JC (2001). A Novel Enhancer of the Apaf1 Apoptosome Involved in Cytochrome c-dependent Caspase Activation and Apoptosis. Journal of Biological Chemistry.

[54] Hlaing T, Guo RF, Dilley KA, Loussia JM, Morrish TA, Shi MM, Vincenz C, Ward PA (2001). Molecular Cloning and Characterization of DEFCAP-L and -S, Two Isoforms of a Novel Member of the Mammalian Ced-4 Family of Apoptosis Proteins. Journal of Biological Chemistry.

[55] Walker JC, Harland RM (2009). microRNA-24a is required to repress apoptosis in the developing neural retina. Genes & Development.

[56] Floyd DH, Zhang Y, Dey BK, Kefas B, Breit H, Marks K, Dutta A, Herold-Mende C, Synowitz M, Glass R, Abounader R, Purow BW (2014). Novel Anti-Apoptotic MicroRNAs 582-5p and 363 Promote Human Glioblastoma Stem Cell Survival via Direct Inhibition of Caspase 3, Caspase 9, and Bim. PLoS ONE.

[57] Shang J, Yang F, Wang Y, Wang Y, Xue G, Mei Q, Wang F, Sun S (2014). MicroRNA-23a Antisense Enhances 5-Fluorouracil Chemosensitivity Through APAF-1/Caspase-9 Apoptotic Pathway in Colorectal Cancer Cells. Journal of Cellular Biochemistry.

[58] Martin MC, Allan LA, Mancini EJ, Clarke PR (2008). The Docking Interaction of Caspase-9 with ERK2 Provides a Mechanism for the Selective Inhibitory Phosphorylation of Caspase-9 at Threonine 125. Journal of Biological Chemistry.

[59] Seifert A, Allan LA, Clarke PR (2008). DYRK1A phosphorylates caspase 9 at an inhibitory site and is potently inhibited in human cells by harmine. FEBS Journal.

[60] Seifert A, Clarke PR (2009). p38α- and DYRK1A-dependent phosphorylation of caspase-9 at an inhibitory site in response to hyperosmotic stress. Cellular Signalling.

[61] Laguna A, Aranda S, Barallobre MJ, Barhoum R, Fernández E, Fotaki V, Delabar JM, de la Luna S, de la Villa P, Arbonés ML (2008). The Protein Kinase DYRK1A Regulates Caspase-9-Mediated Apoptosis during Retina Development. Developmental Cell.

[62] Allan LA, Clarke PR (2007). Phosphorylation of Caspase-9 by CDK1/Cyclin B1 Protects Mitotic Cells against Apoptosis. Molecular Cell.

[63] Deveraux QL, Takahashi R, Salvesen GS, Reed JC (1997). X-linked IAP is a direct inhibitor of cell-death proteases. Nature.

[64] Bratton S, Lewis J, Butterworth M, Duckett C, Cohen G (2002). XIAP inhibition of caspase-3 preserves its association with the Apaf-1 apoptosome and prevents CD95-and Bax-induced apoptosis. Cell death and differentiation.

[65] Mannick JB, Schonhoff C, Papeta N, Ghafourifar P, Szibor M, Fang K, Gaston B (2001). S-Nitrosylation of mitochondrial caspases. The Journal of Cell Biology.

[66] Sakai T, Liu L, Teng X, Mukai-Sakai R, Shimada H, Kaji R, Mitani T, Matsumoto M, Toida K, Ishimura K, Shishido Y, Mak TW, Fukui K (2004). Nucling Recruits Apaf-1/Pro-caspase-9 Complex for the Induction of Stress-induced Apoptosis. Journal of Biological Chemistry.

[67] Venables JP (2004). Aberrant and Alternative Splicing in Cancer. Cancer Research.

[68] Seol DW, Billiar TR (1999). A Caspase-9 Variant Missing the Catalytic Site Is an Endogenous Inhibitor of Apoptosis. Journal of Biological Chemistry.

[69] Srinivasula SM, Ahmad M, Guo Y, Zhan Y, Lazebnik Y, Fernandes-Alnemri T, Alnemri ES (1999). Identification of an Endogenous Dominant-Negative Short Isoform of Caspase-9 That Can Regulate Apoptosis. Cancer Research.

[70] Vu NT, Park MA, Shultz JC, Goehe RW, Hoeferlin LA, Shultz MD, Smith SA, Lynch KW, Chalfant CE (2013). hnRNP U Enhances Caspase-9 Splicing and Is Modulated by AKT-dependent Phosphorylation of hnRNP L. Journal of Biological Chemistry.

[71] Goehe RW, Shultz JC, Murudkar C, Usanovic S, Lamour NF, Massey DH, Zhang L, Camidge DR, Shay JW, Minna JD, Chalfant CE (2010). hnRNP L regulates the tumorigenic capacity of lung cancer xenografts in mice via caspase-9 pre-mRNA processing. The Journal of Clinical Investigation.

[72] Shultz JC, Goehe RW, Wijesinghe DS, Murudkar C, Hawkins AJ, Shay JW, Minna JD, Chalfant CE (2010). Alternative Splicing of Caspase 9 Is Modulated by the Phosphoinositide 3-Kinase/Akt Pathway via Phosphorylation of SRp30a. Cancer Research.

[73] Chalfant CE, Rathman K, Pinkerman RL, Wood RE, Obeid LM, Ogretmen B, Hannun YA (2002). De Novo Ceramide Regulates the Alternative Splicing of Caspase 9 and Bcl-x in A549 Lung Adenocarcinoma Cells: Dependence on protein phosphatase-1. Journal of Biological Chemistry.

[74] Massiello A, Chalfant CE (2006). SRp30a (ASF/SF2) regulates the alternative splicing of caspase-9 pre-mRNA and is required for ceramide-responsiveness. Journal of Lipid Research.

[75] Shultz JC, Goehe RW, Murudkar CS, Wijesinghe DS, Mayton EK, Massiello A, Hawkins AJ, Mukerjee P, Pinkerman RL, Park MA, Chalfant CE (2011). SRSF1 Regulates the Alternative Splicing of Caspase 9 Via A Novel Intronic Splicing Enhancer Affecting the Chemotherapeutic Sensitivity of Non-Small Cell Lung Cancer Cells. Molecular Cancer Research.

[76] Di Stasi A, Tey SK, Dotti G, Fujita Y, Kennedy-Nasser A, Martinez C, Straathof K, Liu E, Durett AG, Grilley B, Liu H, Cruz CR, Savoldo B (2011). Inducible Apoptosis as a Safety Switch for Adoptive Cell Therapy. New England Journal of Medicine.

[77] Straathof KC, Pulè MA, Yotnda P, Dotti G, Vanin EF, Brenner MK, Heslop HE, Spencer DM, Rooney CM (2005). An inducible caspase 9 safety switch for T-cell therapy. Blood.

[78] Bonini C, Ferrari G, Verzeletti S, Servida P, Zappone E, Ruggieri L, Ponzoni M, Rossini S, Mavilio F, Traversari C, Bordignon C (1997). HSV-TK Gene Transfer into Donor Lymphocytes for Control of Allogeneic Graft-Versus-Leukemia. Science.

[79] Tiberghien P, Ferrand C, Lioure B, Milpied N, Angonin R, Deconinck E, Certoux JM, Robinet E, Saas P, Petracca B, Juttner C, Reynolds CW, Longo DL (2001). Administration of herpes simplex-thymidine kinase-expressing donor T cells with a T-cell-depleted allogeneic marrow graft. Blood.

[80] Freytag SO, Khil M, Stricker H, Peabody J, Menon M, DePeralta-Venturina M, Nafziger D, Pegg J, Paielli D, Brown S, Barton K, Lu M, Aguilar-Cordova E (2002). Phase I Study of Replication-competent Adenovirus-mediated Double Suicide Gene Therapy for the Treatment of Locally Recurrent Prostate Cancer. Cancer Research.

[81] Introna M, Barbui AM, Bambacioni F, Casati C, Gaipa G, Borleri G, Bernasconi S, Barbui T, Golay J, Biondi A, Rambaldi A (2000). Genetic Modification of Human T Cells with CD20: A Strategy to Purify and Lyse Transduced Cells with Anti-CD20 Antibodies. Human Gene Therapy.

[82] Belshawl PJ, Spencer DM, Crabtree GR, Schreiber SL (1996). Controlling programmed cell death with a cyclophilincyclosporin-based chemical inducer of dimerization. Chemistry & Biology.

[83] MacCorkle RA, Freeman KW, Spencer DM Synthetic activation of caspases: Artificial death switches. Proceedings of the National Academy of Sciences.

[84] Spencer DM, Belshaw PJ, Chen L, Ho SN, Randazzo F, Crabtree GR, Schreiber SL (1996). Functional analysis of Fas signaling in vivo using synthetic inducers of dimerization. Current Biology.

[85] Tey SK (2014). Adoptive T-cell therapy: adverse events and safety switches. Clin Trans Immunol.

[86] Marin V, Cribioli E, Philip B, Tettamanti S, Pizzitola I, Biondi A, Biagi E, Pule M (2012). Comparison of Different Suicide-Gene Strategies for the Safety Improvement of Genetically Manipulated T Cells. Human Gene Therapy Methods.

[87] Tey SK, Dotti G, Rooney CM, Heslop HE, Brenner MK (2007). Inducible Caspase 9 Suicide Gene to Improve the Safety of Allodepleted T Cells after Haploidentical Stem Cell Transplantation. Biology of Blood and Marrow Transplantation.

[88] de Witte MA, Jorritsma A, Swart E, Straathof KC, de Punder K, Haanen JB, Rooney CM, Schumacher TN (2008). An Inducible Caspase 9 Safety Switch Can Halt Cell Therapy-Induced Autoimmune Disease. The Journal of Immunology.

[89] Hoyos V, Savoldo B, Quintarelli C, Mahendravada A, Zhang M, Vera J, Heslop HE, Rooney CM, Brenner MK, Dotti G (2010). Engineering CD19-specific T lymphocytes with interleukin-15 and a suicide gene to enhance their anti-lymphoma/leukemia effects and safety. Leukemia.

[90] Zhou X, Di Stasi A, Tey SK, Krance RA, Martinez C, Leung KS, Durett AG, Wu MF, Liu H, Leen AM, Savoldo B, Lin YF, Grilley BJ (2014). Long-term outcome after haploidentical stem cell transplant and infusion of T cells expressing the inducible caspase 9 safety transgene. Blood.

[91] Oudejans JJ, Muris JJF, Meijer CJ (2005). Inhibition of Caspase 9 and Not Caspase 8 Mediated Apoptosis May Determine Clinical Response to Chemotherapy in Primary Nodal Diffuse Large B-Cell Lymphomas. Cell Cycle.

[92] Wu GS, Ding Z (2002). Caspase 9 is required for p53-dependent apoptosis and chemosensitivity in a human ovarian cancer cell line. Oncogene.

[93] D'Sa-Eipper C, Leonard JR, Putcha G, Zheng TS, Flavell RA, Rakic P, Kuida K, Roth KA (2001). DNA damage-induced neural precursor cell apoptosis requires p53 and caspase 9 but neither Bax nor caspase 3. Development.

